# Drainage, irrigation and fibrinolytic therapy (DRIFT) for posthaemorrhagic ventricular dilatation: 10-year follow-up of a randomised controlled trial

**DOI:** 10.1136/archdischild-2019-318231

**Published:** 2020-07-04

**Authors:** Karen Luyt, Sally L Jary, Charlotte L Lea, Grace J. Young, David E Odd, Helen E Miller, Grazyna Kmita, Cathy Williams, Peter S Blair, William Hollingworth, Michelle Morgan, Adam P Smith-Collins, Steven Walker-Cox, Kristian Aquilina, Ian Pople, Andrew G Whitelaw

**Affiliations:** 1 Neonatal Neurology, Bristol Medical School, Faculty of Health Sciences, University of Bristol, Bristol, UK; 2 Neonatal Intensive Care Unit, St Michael's Hospital, University Hospitals Bristol NHS Foundation Trust, Bristol, UK; 3 Population Health Sciences, Bristol Medical School, University of Bristol, Bristol, UK; 4 Bristol Randomised Trials Collaboration (BRTC), Bristol Trials Centre, Bristol Medical School, University of Bristol, Bristol, UK; 5 Neonatal Intensive Care Unit, Southmead Hospital, North Bristol NHS Trust, Westbury on Trym, Bristol, UK; 6 Faculty of Psychology, University of Warsaw, Warszawa, Poland; 7 Ophthalmology, Bristol Eye Hospital, University Hospitals Bristol NHS Foundation Trust, Bristol, UK; 8 Bristol Medical School, Faculty of Health Sciences, University of Bristol, Bristol, UK; 9 Child Psychology, Community Children’s Health Partnership, Bristol, UK; 10 Department of Neurosurgery, Great Ormond Street Hospital for Children NHS Foundation Trust, London, UK; 11 Paediatric Neurosurgery, University Hospitals Bristol NHS Foundation Trust, Bristol, UK

**Keywords:** preterm, neonatal, intraventricular haemorrhage, post-haemorrhagic ventricular dilatation, neurodevelopment

## Abstract

**Background:**

Progressive ventricular dilatation after intraventricular haemorrhage (IVH) in preterm infants has a very high risk of severe disability and death. Drainage, irrigation and fibrinolytic therapy (DRIFT), in a randomised controlled trial (RCT), reduced severe cognitive impairment at 2 years.

**Objective:**

To assess if the cognitive advantage of DRIFT seen at 2 years persisted until school age.

**Participants:**

The RCT conducted in four centres recruited 77 preterm infants with IVH and progressive ventricular enlargement over specified measurements. Follow-up was at 10 years of age.

**Intervention:**

Intraventricular injection of a fibrinolytic followed by continuous lavage, until the drainage was clear, and standard care consisting of control of expansion by lumbar punctures and if expansion persisted via a ventricular access device.

**Primary outcome:**

Cognitive quotient (CQ), derived from the British Ability Scales and Bayley III Scales, and survival without severe cognitive disability.

**Results:**

Of the 77 children randomised, 12 died, 2 could not be traced, 10 did not respond and 1 declined at 10-year follow-up. 28 in the DRIFT group and 24 in the standard treatment group were assessed by examiners blinded to the intervention. The mean CQ score was 69.3 (SD=30.1) in the DRIFT group and 53.7 (SD=35.7) in the standard treatment group (unadjusted p=0.1; adjusted p=0.01, after adjustment for the prespecified variables sex, birth weight and IVH grade). Survival without severe cognitive disability was 66% in the DRIFT group and 35% in the standard treatment group (unadjusted p=0.019; adjusted p=0.003).

**Conclusion:**

DRIFT is the first intervention for posthaemorrhagic ventricular dilatation to objectively demonstrate sustained cognitive improvement.

**Trial registration number:**

ISRCTN80286058.

**What is already known on this topic?**

- Progressive ventricular dilatation after intraventricular haemorrhage (IVH) in preterm infants has a very high risk of severe disability and death.
- Several interventions tested in randomised controlled trials (RCTs) have failed to reduce neurodisability in preterm infants with IVH and posthaemorrhagic ventricular dilatation (PHVD).
- Drainage, irrigation and fibrinolytic therapy (DRIFT), in an RCT, reduced severe cognitive disability at 2 years of age.

**What this study adds?**

- DRIFT achieved a sustained reduction of severe cognitive disability at school age, the first intervention for IVH with PHVD to objectively demonstrate long-term benefit.
- The findings are applicable to preterm infants with PHVD in well-resourced healthcare settings.
- The proof of principle that secondary brain injury is reduced by washing away the harmful debris of IVH in a controlled way has been established.

## Introduction

Intraventricular haemorrhage (IVH) remains one of the most serious complications of preterm birth. It is the most common cause of brain injury in preterm infants^1^ and the primary risk factor for special educational needs at school age.^2^ Large IVHs cause a progressive obliterative arachnoiditis, disturbing the flow and absorption of cerebrospinal fluid (CSF),^3^ thereby causing posthaemorrhagic ventricular dilatation (PHVD). Around 70% of very low birthweight (VLBW) infants with severe-grade IVH develop persistent PHVD, and a third of infants with PHVD require surgical drainage of CSF by permanent ventriculoperitoneal (VP) shunt to control ventricular expansion.^4^ The raised pressure, distortion, and neurotoxic and inflammatory effects of blood in the ventricular system cause progressive brain injury and subsequent neurodisability, which is often severe.^5^ Infants under 1000 g who went on to shunt surgery had very high rates of motor, cognitive and multiple disabilities.^6^

A European survey found the most common approach in VLBW infants with PHVD was repeated lumbar punctures (LPs) followed by insertion of a ventricular access device (VAD) to enable regular tapping of CSF and control of ventricular expansion. If the need for tapping persists, a VP shunt is then inserted weeks later when the blood and protein have cleared from the CSF and the infant’s weight has reached 2 kg.^7^ VAD and VP shunt insertion are associated with significant risk of infection and malfunction.^8^

Several interventions tested in randomised controlled trials (RCTs) have failed to reduce neurodisability rates as a result of PHVD.^9^ Drainage, irrigation and fibrinolytic therapy (DRIFT)^10–12^ was developed due to the unsatisfactory results of other treatments. The objective is to remove proinflammatory cytokines, free iron and old blood from within the ventricles, and reduce both pressure and distortion.

The DRIFT RCT was conducted between 2003 and 2006.^10^ There were no differences in the need for VP shunt or death at 6 months. However, at 2 years post-term, severe disability or death was significantly reduced in the DRIFT group.^11^

We have followed up these patients at 10 years of age to determine if the cognitive advantage seen with DRIFT at 2 years continued through to school age. The secondary objectives were to assess long-term visual, sensorimotor function and emotional/behavioural difficulties. We hypothesised that DRIFT would reduce severe cognitive disability at school age.

## Methods

### Initial study

DRIFT is a surgical approach developed in Bristol. Temporary frontal and left occipital ventricular catheters are inserted under anaesthesia. Tissue plasminogen activator is injected intraventricularly at a subsystemic dose. The ventricles are irrigated by artificial CSF through a frontal catheter. When the ventricular system has been cleared of blood and debris, catheters are removed.

After feasibility testing showed DRIFT was technically possible and promising,^12^ the DRIFT RCT started recruiting in 2003.^10^

Eligible babies were preterm, had had IVH and had expanded cerebral ventricles over predetermined measurements. In total 77 babies (54 in Bristol (UK), 20 in Katowice (Poland), 2 in Glasgow (UK) and 1 in Bergen (Norway)) were randomised during 2003–2006 to either DRIFT or standard treatment, which consisted of LPs to control excessive expansion and pressure symptoms. If repeated LPs were needed, a VAD was surgically inserted to facilitate tapping of CSF. Every infant randomised to DRIFT received DRIFT, and no infant in the standard treatment group received DRIFT. At the time of intervention it was not possible to blind clinicians; however, all subsequent researchers were unaware of treatment group allocation. Full details of the trial have been published.^10^

Of the 77 babies randomised, 69 survived until 2 years. Severe cognitive disability (Bayley Mental Development Index (MDI) 3 SD below the mean) was 31% in the DRIFT group and 59% in the standard treatment group (adjusted OR: 0.17 (95% CI 0.05 to 0.57)), and the difference in median MDI score was more than 18 points.^11^

### Follow-up study

The follow-up study was designed with input from children and parents who had taken part in the initial feasibility study. Assessments consisted of cognitive, motor and visual ability, presence and severity of cerebral palsy (CP), and parental completion of a vision and behavioural inventory. Children in Poland did not have visual or motor assessments. All assessments were performed between February 2015 and April 2016. All outcome assessors were blinded to treatment allocation.

The primary hypothesis was whether DRIFT would reduce severe cognitive disability in children assessed at school age.

Imbalances in covariates affecting cognition were seen at randomisation and in the previous 2-year follow-up. Consequently, for this work we prespecified in the statistical analysis plan adjustment for birth weight, IVH grade and sex, with approval by the independent National Institute for Health Research follow-up study steering committee (including an independent statistician). The secondary outcomes were also adjusted for age, as the follow-up ages ranged from 8 to 12 years. Primary, secondary and exploratory analyses described here were prespecified in the study protocol (https://www.journalslibrary.nihr.ac.uk/programmes/hta/123561/%23/).

The follow-up study was funded by the National Institute of Health Research (Health Technology Assessment), for which a report/monograph has been published in full.^13^

### Primary outcome

#### Cognitive disability at school age

Cognitive assessments were undertaken by two child psychologists. The British Ability Scales-III was used for children with a developmental age of 3+ years.^14^ For children who did not meet this threshold, the Bayley Scales of Infant and Toddler Development-III was administered.^15^ The final scores were in the format of a cognitive quotient (CQ; from 0 to 100+), based on the division of their developmental age equivalent by their actual age (multiplied by 100).^16^ The primary analysis was based on the cognitive scores of surviving children, although a sensitivity analysis was performed including children who died (due to disability), where the CQ for these children could reasonably be assumed to be 0. Several sensitivity analyses were carried out, including the use of a binary outcome (death or severe cognitive disability), where all deaths over the 10-year period and deaths post 2 years that were deemed related to disability were included as negative outcomes. For patients who died after the 2-year follow-up, where the cause of death was unknown, we assumed that their death was due to disability if they had severe disability at 2 years. Severe cognitive disability was defined as a cognitive score below 3 SD of the population mean.

### Secondary outcomes

#### Cerebral visual function

For the main visual outcomes, parents were asked about their child’s vision, describing them as ‘No concerns’, ‘Normal with Correction’, ‘Useful but not fully correctable’ and ‘Blind or perceives light only’. A binary outcome was created that split these into good visual outcome (no concerns/normal with correction) and poor visual outcome (useful but not fully correctable/blind or perceives light only). Parents were asked 23 questions about their child’s visual behaviour from an inventory identifying examples of vision processing impairment.^17^ A mean score was created.

#### Sensorimotor disability

Children were assessed using the Movement Assessment Battery for Children-2 (MABC-2) by a paediatric physiotherapist.^18^ The presence and severity of CP were also compared between the two groups according to the recommendations of the Surveillance of Cerebral Palsy in Europe, using the Gross Motor Function Classification System.^19^ All children with CP were classified as having severe sensorimotor disability, moderate if MABC-2 scores were between 57 and 67, and none if MABC-2 >67.

#### Emotional/behavioural function

Parents were asked to complete the Strengths and Difficulties Questionnaire (SDQ), which assesses child behaviour. The final score classifies the child as having normal or abnormal behaviour.^20^

### Power calculation

Based on the effect size of DRIFT treatment on severe cognitive disability at 2 years,^11^ a two-group continuity-corrected χ^2^ test with 5% two-sided significance level would have 80% power to detect the difference in severe cognitive disability between a standard treatment group proportion of 59% and OR of 0.17 (ie, an intervention proportion of 19.7%) when the sample size in each group is 28. Assuming 90% follow-up rate, 60 infants (30 in each group) would have 97% power (with an alpha of 5%) to detect a mean cognitive score difference of 1 SD (commonly 15 points) between the DRIFT and the standard treatment group.

### Statistical analysis

All statistical analyses were performed using STATA V.14.1 software. All p values were two-sided and considered significant if p<0.05.

## Results

### Study population and participant flow

**Figure 1:**
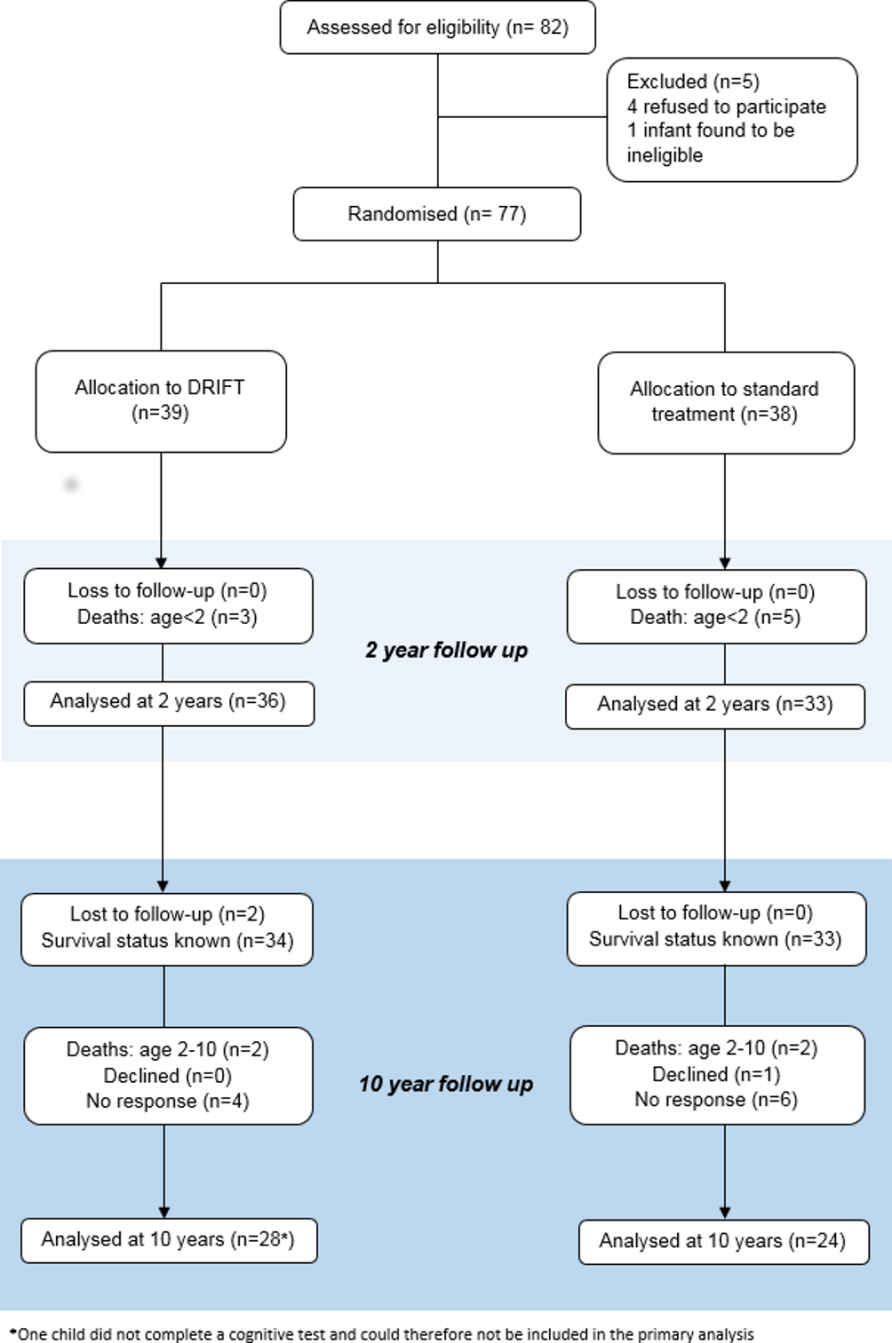
Drainage, irrigation and fibrinolytic therapy (DRIFT) participant flow.

Figure 1 shows the layout of the trial and the different levels of dropout and analyses. Seventy-seven babies were originally recruited to either receive DRIFT (39 infants) or standard treatment (38 infants).

At 2-year follow-up there had been eight deaths due to complications of prematurity, but no loss to follow-up. Approximately 8 years later (between September 2015 and April 2016), parents were contacted and asked to take part in the 10-year follow-up study. Two patients (both in the DRIFT group) could not be traced. This left 67 patients where the survival status was known. Of these, there were 2 deaths in the DRIFT group, 2 deaths in the standard treatment group (all 4 were associated with severe disability), 1 patient who declined to take part in the follow-up (standard treatment group) and 10 who gave no response (4 DRIFT, 6 standard), leaving 52 available for assessment (28 DRIFT, 24 standard). For the primary outcome we obtained 51 children’s CQ score: 27 in the DRIFT group and 24 in the standard treatment group.

### Baseline data

For the 52 children available for follow-up assessments at 10 years, there were imbalances seen in sex and birth weight (table 1). In the DRIFT group, 22 (79%) children were male, whereas in the standard treatment group 63% were male. The mean birth weight was 1322 g in the standard treatment group and 1102 g in the DRIFT group.

**Table 1:** Characteristics of participants assessed at 10 years by trial allocation

	n	DRIFT		n	Standard	
		Mean (SD) or n (%)			Mean (SD) or n (%)	
Total number of participants	28			24		
Centre						
Bristol, UK	28	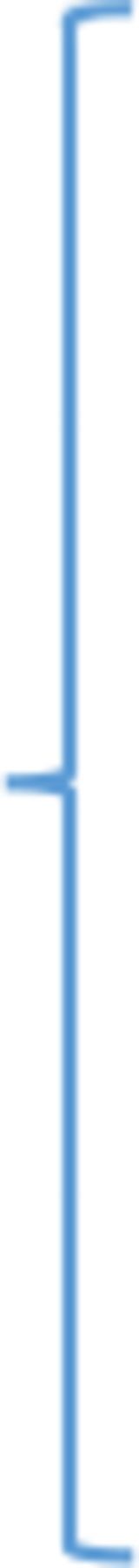	23 (82%)	24	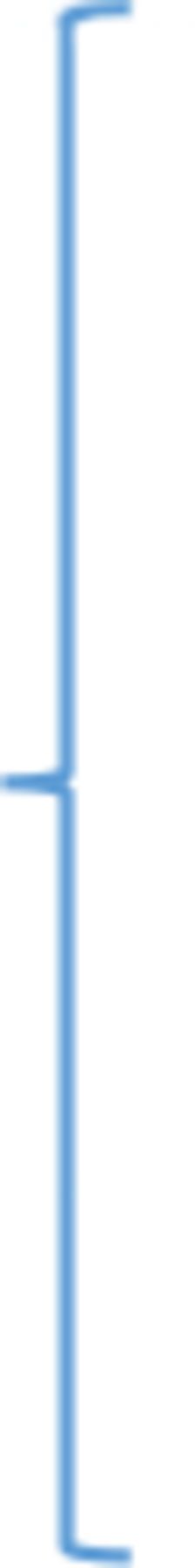	19 (79%)
Katowice, Poland			3 (11%)			4 (17%)
Glasgow, UK			1 (4%)			1 (4%)
Bergen, Norway			1 (4%)			0 (0%)
Sociodemographics at birth						
Age at randomisation (days)	28	18.68 (5.00)	24	19.17 (4.53)
Sex: male*	28	22 (79%)	24	15 (63%)
Clinical characteristics at birth						
Birth weight (g)*	28	1101.89 (335.54)	24	1322.46 (534.68)
Gestation (weeks)	28	27.64 (2.56)	24	28.50 (3.05)
Grade of IVH: 4	28	14 (50%)	24	11 (46%)
Maternal age at birth	14	28.50 (6.99)	12	28.17 (6.32)
Median IMD 2010, postcode at birth† (IQR)	18	26.7 (8.2–36.5)	18	27.6 (11.2–45.8)
Measures at 2 years						
Experienced second IVH*	28	8 (29%)	24	3 (13%)
VP shunt	28	11 (39%)	24	8 (33%)
Ventricular access device*	28	13 (46%)	24	19 (79%)
Measures at 10 years						
Age at 10-year assessment (years)	28	10.56 (1.07)	24		10.76 (1.06)
Weight (kg)	28	35.41 (10.05)	23	34.73 (10.51)
Height (cm)	28	139.09 (12.22)	23	142.26 (11.34)
Head circumference (cm)	28	52.88 (2.53)	23	52.00 (3.43)
Maternal education*						
Left school at 16	28	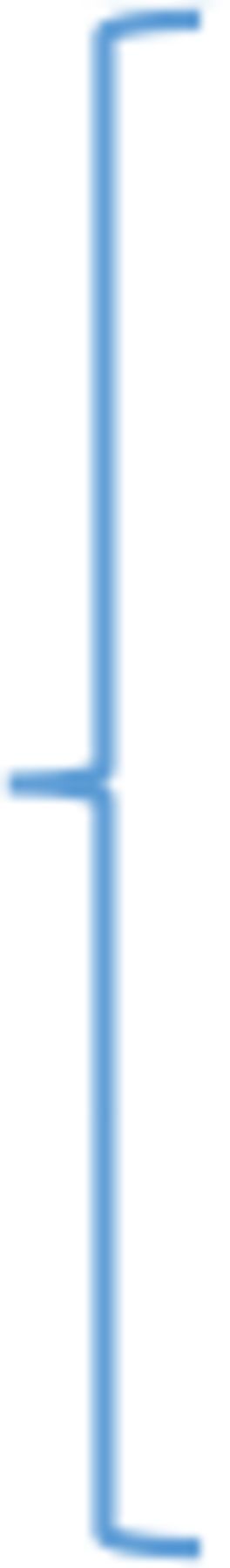	10 (36%)	23	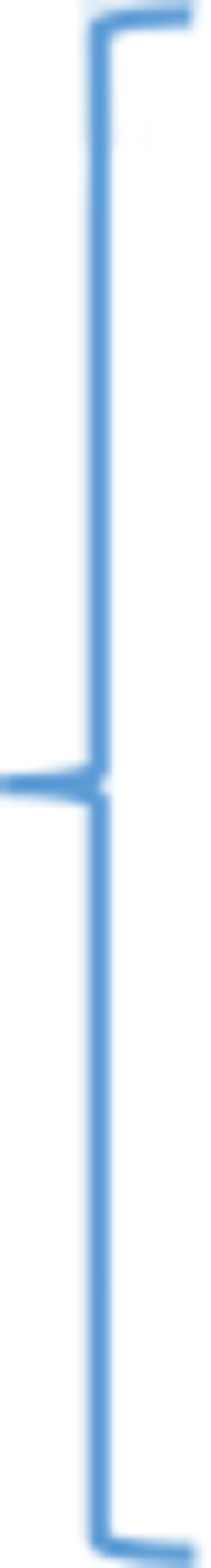	11 (48%)
Further education			6 (21%)			5 (22%)
University degree			12 (43%)			7 (30%)

*Difference of 10%/0.5 SD or higher between the groups.

†English Index of Multiple Deprivation (IMD) 2010 scores, UK Data Service Census Support (http://geoconvert.mimas.ac.uk/). Higher scores indicate higher levels of deprivation. IMD based on the children’s home postcode at birth for those residing in England only.

DRIFT, drainage, irrigation and fibrinolytic therapy; IVH, intraventricular haemorrhage; VP, ventriculoperitoneal.

### Primary analyses

#### Primary outcomes

**Figure 2:**
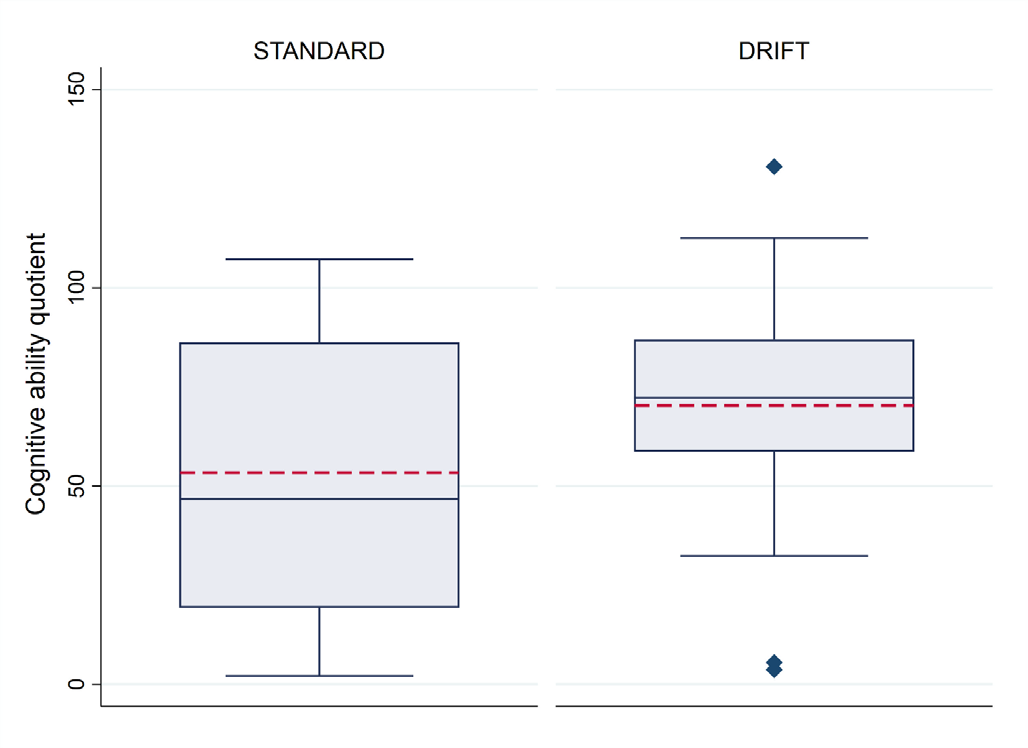
Cognitive quotient, by trial allocation. The box plot indicates the median (central blue line in the box), mean (red dashed line), 25th percentile (bottom line of the box), 75th percentile (top line of the box), and the whiskers for the minimum and maximum values (excluding outliers). The diamonds beyond these whiskers are the outliers, which are observations that lie at an abnormal distance from other values. Outliers are defined as those higher than 1.5× IQR+75th percentile or lower than 25th percentile−1.5× IQR. DRIFT, drainage, irrigation and fibrinolytic therapy.

CQ scores were relatively normally distributed with an overall mean of 62.0 and a median of 68.7. Figure 2 shows the distribution of scores by treatment allocation. Children receiving DRIFT had a mean quotient score of 69.3 (median 72.3) vs 53.7 (median 44.6) for children receiving standard treatment. The maximum CQ score was 130.6 (DRIFT group), indicating a cognitive ability that is 30% higher than we would expect to see at their age (developmental age 30% higher than actual age). The highest CQ score achieved in the standard treatment group was 107.2. Only two children had CQ score below 30 (profound cognitive disability) in the DRIFT group, compared with seven children in the standard treatment group.

Table 2 shows the results of the primary analysis, including and excluding deaths. Given the larger than expected attrition/death rate, precision was lower than predicted, exacerbated further by the large SD for the CQ. Despite this, the results are in parallel with those at 2 years, with crude estimates giving weak evidence that the DRIFT intervention increases cognitive ability at 10 years (p=0.096). After adjusting for the prespecified variables sex, birth weight and IVH grade, this evidence was strengthened and indicated that children in the DRIFT group had, on average, CQ scores 23.47 points higher than those who received standard treatment (p=0.009). This translates into a developmental cognitive advantage of 2.5 years (95% CI 0.5 to 4.4).

**Table 2:** Primary outcome

Variable	n (DRIFT:standard)	DRIFT Mean (SD) or n (%)	Standard Mean (SD) or n (%)	Crude difference (95% CI), p value	Adjusted difference (95% CI), p value*
Primary outcome					
Cognitive ability quotient	27:24	69.33 (30.06)	53.68 (35.70)	15.65 (−2.86 to 34.16), 0.096†	23.47 (6.23 to 40.71), 0.009†
Sensitivity analyses					
Cognitive ability quotient‡	29:26	64.55 (34.04)	49.55 (37.22)	15.00 (−4.28 to 34.27), 0.125†	22.33 (4.77 to 39.89), 0.014†
Alive and without severe cognitive disability§	29:26	21 (72%)	11 (42%)	3.58 (1.16 to 11.04), 0.026¶	9.96 (2.12 to 46.67), 0.004¶
Alive and without severe cognitive disability**	32:31	21 (66%)	11 (35%)	3.47 (1.23 to 9.78), 0.019¶	7.69 (1.96 to 30.11), 0.003¶

*Adjusted for sex, birth weight and grade of IVH.

†Linear regression (difference in means).

‡Giving children who have died post 2 years a score of 0.

§Including all 4 deaths after 2 years as a negative outcome.

¶Logistic regression (OR).

**Including all 12 deaths as a negative outcome (akin to the study results at 2 years).

DRIFT, drainage, irrigation and fibrinolytic therapy; IVH, intraventricular haemorrhage.

#### Sensitivity analyses

The binary outcome used in the 2-year follow-up was replicated at 10 years, resulting in 21 of 32 (66%) patients surviving without severe cognitive disability in the DRIFT group compared with 11 of 31 (35%) patients in the standard treatment group (adjusted OR 7.69 (1.96 to 30.11), p=0.003). Survival without severe cognitive disability (removing deaths before 2 years) also gave very similar results to the continuous CQ outcome (unadjusted OR 3.58 (1.16 to 11.04), p=0.026; adjusted OR 9.96 (2.12 to 46.67), p=0.004). The number needed to treat (NNT) to prevent one death or case of severe cognitive disability was 3. More details on cause of death and sensitivity analyses are included in the online supplementary appendix.

10.1136/archdischild-2019-318231.supp1 Supplementary data

### Secondary outcomes

#### Parent-reported vision

A higher proportion of children from the DRIFT group had ‘good visual outcome’, but there was high variance and this was not statistically significant (adjusted OR 3.73 (95% CI 0.66 to 21.14), p=0.136). A small difference was found in the question inventory mean score (adjusted mean difference −0.12 (95% CI −0.47 to 0.24), p=0.502) (table 3).

**Table 3:** Secondary outcomes

	n (DRIFT:standard)	DRIFT n (%) or mean (SD)	Standard n (%) or mean (SD)	Unadjusted difference (95% CI), p value	Adjusted difference* (95% CI), p value
Parent-reported visual function					
Good vision	27:24	23 (85%)	17 (71%)	2.37 (0.60 to 9.40), 0.22†	3.73 (0.66 to 21.14), 0.14†
Questionnaire mean score	28:21	4.50 (0.70)	4.65 (0.38)	−0.15 (−0.49 to 0.19), 0.38‡	−0.12 (−0.47 to 0.24), 0.50‡
Sensorimotor disability					
None		2 (7%)	3 (14%)		
Moderate	27:21	2 (7%)	2 (10%)	0.55 (0.13 to 2.34), 0.42§	3.66 (0.33 to 40.34), 0.29§
Severe		23 (85%)	16 (76%)		
Cerebral palsy					
Diagnosed with cerebral palsy (%)	28:24	17 (61%)	14 (58%)	1.10 (0.36 to 3.35), 0.86†	0.37 (0.07 to 2.00), 0.25†
GMFCS level 1		7 (41%)	5 (36%)		
GMFCS level 2		4 (24%)	3 (21%)		
GMFCS level 3		2 (12%)	0 (0%)		
GMFCS level 4		0 (0%)	2 (14%)		
GMFCS level 5		4 (24%)	4 (29%)		
Ambulant¶		11 (65%)	8 (57%)	1.38 (0.32 to 5.88), 0.67	1.32 (0.24 to 7.25), 0.75
Strengths and Difficulties Questionnaire					
Total score	28:22	14.89 (8.48)	13.36 (6.59)	1.53 (−2.89 to 5.94), 0.49‡	2.01 (−2.78 to 6.81), 0.40‡

*Adjusted for age, sex, birth weight and grade of IVH.

†Logistic regression (OR).

‡Linear regression (difference in means).

§Ordinal logistic regression (OR per increase in category).

¶Children with cerebral palsy were categorised as ambulant (GMFCS level 1–2) or non-ambulant (GMFCS level 3–5).

DRIFT, drainage, irrigation and fibrinolytic therapy; GMFCS, Gross Motor Function Classification System; IVH, intraventricular haemorrhage.

#### Sensorimotor disability

There was no difference in sensorimotor disability, with severe, moderate and no sensorimotor disability percentages of 85%, 7% and 7% for patients in the DRIFT group, respectively, compared with 76%, 10% and 14% in the standard treatment group (adjusted OR 3.66 (0.33 to 40.34), p=0.290). There was no significant difference in the percentages with CP or severity of CP between the two groups (61% vs 58% for DRIFT and standard treatment, respectively) (table 3).

#### Emotional/behavioural function

For the SDQ total score, higher values indicated more ‘abnormal’ behaviour. There was no difference between the two groups (adjusted mean difference 2.01 (95% CI −2.78 to 6.81), p=0.401).

### Sensitivity/subgroup analyses

Additional sensitivity analyses, described in the online supplementary appendix, were carried out that include multiple imputation and adjustment for centre, all of which were consistent with the primary outcome (online supplementary eTable 1). We did not find any subgroup effects to suggest that DRIFT was more effective in certain subgroups, although it needs to be emphasised that we were not powered to detect any differences between subgroups (online supplementary eTable 2). Maternal educational attainment was imbalanced at 10 years, with a greater proportion of mothers in the DRIFT group accessing university education (table 1). In a post-hoc sensitivity analysis, we adjusted for maternal level of education (online supplementary eTable 1). Adjustment for maternal education (measured 10 years after randomisation) resulted in a slight attenuation of effect size with an 11.5 point CQ advantage (unadjusted p=0.2) and a significant 20.1 point CQ advantage after adjustment for the prespecified variables sex, birth weight and IVH grade (adjusted p=0.02) with DRIFT.

Additional data from parental history were available regarding special education needs in children. After adjustment, those in the DRIFT group showed a trend towards lower odds of special school attendance in the preceding 12 months compared with those in the standard treatment group (OR 0.27 (95% CI 0.07 to 1.05), p=0.059). This suggests that there may be a benefit in terms of educational attainment from DRIFT treatment.

## Discussion

### Summary of findings

DRIFT treatment of preterm infants with severe IVH and PHVD improves cognitive ability at 10-year follow-up when taking into account birth weight, IVH grade and sex. There were no significant differences in the secondary outcomes: parent-reported visual impairment, sensorimotor disability or emotional/behavioural difficulties. Surrogate markers of functional ability (special education) showed a trend towards long-term benefits of DRIFT treatment.

Crucially, infants who received DRIFT were almost twice as likely to survive without severe cognitive disability than those who received standard treatment. While the CIs were wide, the point estimate suggests that the NNT for DRIFT to prevent one death or one case of severe cognitive disability was 3.

### Strengths and limitations

The strength of this study is the long-term follow-up to middle-school age, which strengthens the validity of conclusions around cognitive ability. In neonatal interventional trials, long-term follow-up is challenging as families move. It requires active buy-in from both children and their parents and a significant time commitment for families. Where a significant proportion of survivors have severe neurodisabilities, as seen with PHVD, the logistics to return for follow-up assessment become even more challenging.

Where children have a very wide range of abilities, precise cognitive scoring becomes a significant challenge. Our approach to cognitive assessment achieved CQ in children of all abilities. Inclusion of special education as a pragmatic post-hoc outcome gives some idea of the likely gains going forward into an independent adulthood.

Ongoing family involvement and the organisation of the British National Health Service ensured a very high follow-up rate at school age in the UK. Only two patients had an unknown survival status at 10 years, and best and worst case scenarios were also explored (online supplementary eTable 1).

While the higher CQ score results were apparent in all analyses, the effect did not reach conventional levels of statistical significance in the unadjusted analyses of mean CQ scores. However, important characteristics were unbalanced at randomisation and at 10-year follow-up, with a larger proportion of higher risk cases in the DRIFT group. In the DRIFT group, infants were significantly smaller, less mature, with greater proportion of male and had more severe-grade IVH, and consequently adjustments for the imbalances were prespecified in the analysis plan. Of note, the association seen in the binary outcome (survival without severe cognitive disability) was clear in the unadjusted and adjusted analyses. Another limitation was that maternal education was not collected at birth, and our use of a proxy measure of maternal education at 10 years is difficult to interpret, especially if educational attainment was gained after baseline. We have adjusted for maternal educational level, which may be a useful proxy but may not give a full picture of family functioning. Details about use of rehabilitative services were not collected as these data are not formally available in the UK and it was not reasonable to expect families to reliably recall the type and level of intervention over a period of 9–10 years after discharge from the neonatal unit.

The main limitation is reduced precision of results due to the size of the trial. This intervention was innovative and invasive, and for safety reasons the trial had stringent stopping criteria which limited the achieved sample size. This unfortunately resulted in a lower sample size than was required to give 80% power for the primary outcome (CQ), and consequently wide CIs around the point estimates for both the continuous and binary outcomes.

### Interpretation of results

Preterm infants with severe IVH and PHVD have a very high reported rate of neurodisability. The National Institute of Child Health and Development study, the largest of its kind, studied preterm infants with severe IVH (grade 3 and 4), of whom almost 25% had PHVD.^6^ At 18–22 months corrected age, 68% of children with severe-grade IVH and PHVD were reported to have moderate cognitive impairment (MDI below 2 SD) and 41% had severe cognitive impairment (MDI below 3 SD). The presence of haemorrhagic parenchymal infarction, in addition to PHVD, increased the risk of CP to between 80% and 90%.

In our study follow-up at 10 years found similar severe cognitive disability rates: 52% in the standard treatment arm. The proportion of children with severe cognitive disability with DRIFT was 21%. This paper further supports that the DRIFT process improves outcome in this group of infants, with evidence of improvement in the number of infants with a very low cognitive score and an overall increase in the mean cognitive score. With the precision available in this trial, we were unable to identify if DRIFT has a disproportionate effect across this range. Children who received DRIFT were also more likely to attend mainstream schools. The reduction in severe cognitive disability seen with this intervention is likely to translate into the ability to lead more independent lives into adulthood.

DRIFT had an effect on cognition but did not appear to improve motor function. Of the children, 50% (DRIFT) and 46% (standard care) had haemorrhagic parenchymal infarctions (grade 4 IVH). The most likely explanation for the disproportionate effect on cognition is that simple irrigation, although effective at reducing secondary global neurotoxicity and damage to cortical and subcortical tissue, is not sufficient to promote tissue regeneration in critical motor tracts after significant parenchymal infarction. Motor outcome is likely primarily determined by the initial amount of acute tissue injury/loss incurred by a large focal haemorrhagic parenchymal infarction. There was a strong trend towards improved visual function, but this did not reach statistical significance due to marked variability of outcome.

### Implications for practice in the context of current standard treatments and other research

DRIFT is the first and only intervention for PHVD in preterm infants to demonstrate long-term benefit in an RCT. The proof of principle that secondary brain injury is reduced by washing away the harmful debris of IVH in a controlled way with DRIFT has been established. This is a hugely promising development in a group of infants with a high rate of long-term neurodisability.

Existing standard treatment with relief of intracranial pressure by CSF drainage through LP or insertion of a VAD results in worse outcomes than those seen with DRIFT. The recently published data from the Early vs Late Ventricular Intervention Study (ISRCTN43171322), a multicentre RCT comparing two treatment thresholds for VAD insertion after PHVD, have not revealed benefits from commencing standard treatment at a lower threshold. The short-term outcomes show no benefit from the lower threshold and significantly more invasive procedures.^21^ There was no significant difference in mortality or neurodevelopmental outcome at 2 years of age.^22^

Alternative surgical interventions are being tested. A feasibility study of ventricular endoscopic lavage demonstrated fewer short-term complications and need for VP shunts in comparison with standard treatment in historical controls. However, there is a lack of long-term data on outcomes from these children.^23^

DRIFT then offers the only proven option for improving outcomes after PHVD in preterm infants. The aim of DRIFT is to improve neurodevelopmental outcomes, and therefore while there appears to be a net benefit to the infant this would need to be balanced with the potential risks (eg, infection, secondary haemorrhage and rapid fluctuation of intracranial pressure). The expertise required for skilled insertion of two ventricular catheters into a tiny infant, followed by prolonged accurate monitoring of intracranial pressure, adjustment of drainage rate and detection of catheter blockage, all done with immaculate aseptic technique, is significant. The complexity, risks and rarity of candidates are roughly comparable with those of extracorporeal membrane oxygenation, and implementation of DRIFT as a clinical service would likely require a small number of highly specialised centres in order to achieve adequate throughput of patients to develop and maintain such expertise, for example, four or five for the UK. For comparison, Haukeland Hospital in Bergen is the only centre in Norway (population 5.3 million) providing DRIFT service to referred infants.

## Conclusions

PHVD is associated with high levels of severe neurodisability with marked detrimental effect on cognitive function. DRIFT is the first intervention to objectively demonstrate sustained cognitive improvement in preterm infants with PHVD that is sustained into middle-school age. Although invasive and technically demanding, this treatment approach provides clear improvements in outcome and deserves detailed further evaluation.
