# Supplementary material for: Drainage, irrigation and fibrinolytic therapy (DRIFT) for posthaemorrhagic ventricular dilatation: 10-year follow-up of a randomised controlled trial
Source: Arch Dis Child Fetal Neonatal Ed. 2020 Jul 4;105(5):466–73. doi: 10.1136/archdischild-2019-318231 (PMC7547901; doi:10.1136/archdischild-2019-318231)
Supplement: Supplementary data [file fetalneonatal-2019-318231supp001.pdf]

# WEB APPENDIX

## METHODS

### Sensitivity analyses

*Cognitive Quotient*

*Cognitive Quotient for the Bristol cohort*

*Binary outcome: alive and without severe cognitive disability*

*Ordinal outcome: grading of cognitive disability*

*Adjustment for baseline covariates (by more than 10% or 0·5SDs)*

*Adjustment for maternal education*

*Imputation of missing data at ten years*

*Best and worst case scenarios*

### Subgroup analyses

## RESULTS

### Sensitivity analyses

**eTable 1. Sensitivity Outcomes**

**eTable 2. Subgroup Analyses**

## REFERENCES

## METHODS

### Sensitivity analyses

To test the robustness of the primary outcome results, various sensitivity analyses were carried out. As cause of death was difficult to ascertain, different methods were used to ensure that results were consistent; these included:

*Cognitive Quotient*

It had not been anticipated that deaths would occur from disability between two and ten years of age, however, it was considered an important outcome for the trial. Of the four deaths, one child from each group had a certificate of cause of death that confirmed the death was due to disability and one child from each arm did not have traceable certification therefore, based on their developmental scores at two years, their deaths were assumed to be related to their severe disability. As a sensitivity analyses these four children were given a cognitive quotient (CQ) score of zero.

*Cognitive Quotient for the Bristol cohort*

The majority of patients were recruited from the Bristol center and, to check that results remained the same when looking specifically at protocol procedures in a single center, children from Glasgow, Norway and Poland were excluded.

*Binary outcome: alive and without severe cognitive disability*

The two-year outcomes paper [1] separated the children into those who were alive and without sensorimotor or cognitive disability versus those who had died or had severe sensorimotor or cognitive disability. The primary research objective for this follow-up study was cognitive ability, assessed using either the British Ability Scales III (BASIII) [2] or the Bayley III scales (BSIDIII) [3] age equivalent scores (Protocol: https://www.journalslibrary.nihr.ac.uk/programmes/hta/123561/#/). BSIDIII scales were used if a child's developmental age was below three years (i.e. below the range of the BASIII test). However, given the results at two years it was considered important to try and replicate the findings at ten years. Therefore, a binary outcome was created that included all deaths as a negative outcome, along with a General Conceptual Ability (GCA) score of <55.



Luyt K, *et al. Arch Dis Child Fetal Neonatal Ed* 2020; 105:466–473. doi: 10.1136/archdischild-2019-318231

*Ordinal outcome: grading of cognitive disability*

The team felt it was important to differentiate between the different levels of cognitive disability and therefore included this as a sensitivity analysis. We did not anticipate that certain individuals would have GCA scores greater than 85, however, this was true for 11 individuals. Therefore, the categories were expanded (post-hoc) to 1. Deceased, 2. Severe (GCA<55 or Bayley scale used), 3. Moderate (55≤GCA<70), 4. Mild (70≤GCA<85), 5. No cognitive disability (GCA≥85).

*Adjustment for baseline covariates (by more than 10% or 0·5SDs)*

Although sex, birthweight and grade of intraventricular hemorrhage (IVH) had been pre-specified as covariates that were imbalanced at two years the team also wanted to adjust for any imbalance between the arms of those assessed at ten years.

*Adjustment for maternal education*

This was not a pre-specified sensitivity analysis. The team wanted to adjust for maternal education status, as this may have influenced cognitive development after the two-year assessment. However, as this was not collected at baseline, maternal education status at ten years was used as a proxy measure.

*Imputation of missing data at ten years*

Similarly to Biering et al. [4] we chose to carry out five different imputation models that made different assumptions about the data, particularly death. These included each of the four permutations of including an indicator variable for death and/or imputing scores of zero for those who died, as well as a separate imputation model excluding the four deaths. As we did not have any cognitive data on those who died before two years, these were excluded from the imputation models. Baseline variables were assessed to see whether they were predictive of missingness or were appropriate predictors of the primary model, using logistic and linear regression. Any baseline variables associated with the primary outcome of interest or its missingness were added to the imputation model to inform the imputation process. Imputation by chained equations was carried out in STATA 14.1 to create 40 separate imputations that were then combined using Rubin's rules.

*Best and worst case scenarios*

There were two children lost to follow up in the DRIFT group where we were not aware of their survival status. The analysis was repeated, after imputing zeros as a worst case scenario and after imputing median scores for their group as a best case scenario.

**Subgroup analyses**

Interaction tests were added to the primary outcome to see if the effects of the DRIFT intervention were more pronounced in certain subgroups of children; the pre-specified dichotomized subgroups included:

- Gestation (28 weeks and above vs. <28 weeks)
- Grade of IVH (Grade 3 vs. 4)
- Age of randomization (Day 1-20 vs. 21+ days)
- Unilateral vs bilateral dilatation on ultrasound scan at randomization.
- Sex
- Pre- and post-enhanced vigilance in 2006.

Additionally, maternal education was added as a post-hoc sub group analysis, categorized as ‘low’ if the mother left school at 16 years and ‘high’ if the mother carried on with further education post 16 and/or had accessed university education.

**RESULTS**

**Sensitivity analyses**

All sensitivity analyses gave results that were consistent with the primary analysis (eTable 1). Adjustment for center and maternal education (not concurrently) weakened the effect size slightly but models additionally adjusting for sex, birthweight and IVH grade were still in favour of DRIFT. Adjusting for factors that showed imbalance at baseline gave very similar results to the primary adjusted model as the variables imbalanced at baseline were sex and birthweight; two of the three covariates used in the primary model. Results from each of the five multiple imputation models had slightly tighter confidence intervals but similar effect sizes. After imputing scores of zero for those lost to follow up in the DRIFT group as a ‘worst case scenario’, the effect estimate was weakened to produce a treatment difference consistent with chance, however, still in favor of DRIFT. The best case scenario gave similar results to the primary analysis, after imputing scores of zero for those who died post two years.

Results from the binary outcome of death or severe cognitive disability gave even stronger evidence to suggest a benefit from DRIFT. Including all deaths between randomization and ten years as a negative outcome left 21/32 (66%) of children alive and without severe cognitive disability in the DRIFT group, compared with 11/31 (35%) in the standard treatment group; adjusted odds ratio 7·69 (95% CI 1·96, 30·11), p=0·003. For the four deaths that occurred after two years, the trial team confirmed death due to severe disability from the cause of death certification for two children. For the two deaths where certification could not be obtained the assumption was made that the causes of death were also attributable to disability based on their severe disability grading at their two-year assessments. Deaths prior to two-year follow-up were handled with caution as they mainly occurred in the first months of life when accurate ascertainment of presence and extent of disability is difficult. Prior to two years only four certificates of cause of death could be obtained of which three were deemed unrelated to neurological causes (1 DRIFT and 2 standard treatment) and 1 (standard treatment) was attributable to neurological causes. That left four deaths before two years where the cause could not be obtained (2 DRIFT and 2 standard treatment). Excluding deaths before two years of age resulted in 21/29 (72%) of children alive and without cognitive disability in the DRIFT group compared with 11/26 (42%) in the standard treatment group; adjusted odds ratio 9.96 (95% CI 2·12, 46·67), p=0·004. There was no evidence to suggest that there were any subgroup interactions with the treatment effect (eTable 2).



Luyt K, *et al. Arch Dis Child Fetal Neonatal Ed* 2020; 105:466–473. doi: 10.1136/archdischild-2019-318231

**eTable 1. Sensitivity outcomes**

| | n(D:S) | DRIFT n(%)/ *Mean(SD)* | Standard n(%)/ *Mean(SD)* | Unadj. difference (95% C.I); P value | Adj. difference (95% C.I); P value[a] |
|---|---|---|---|---|---|
| ***Cognitive Quotient (CQ; Bristol cohort only)*** | | | | | |
| Score | 23:19 | *71·76 (27·42)* | *57·83 (34·78)* | 13·93 (-5·46, 33·33); 0·15 | 24·88 (6·82, 42·94); 0·008 |
| Score[b] | 24:20 | *68·77 (30·56)* | *54·94 (36·24)* | 13·84 (-6·48, 34·15); 0·18 | 23·27 (4·65, 41·88); 0·016 |
| ***Cognitive disability category*** | | | | | |
| Deceased | | 2 (7%) | 2 (8%) | | |
| Severe disability | | 6 (21%) | 13 (50%) | | |
| Moderate disability | 29:25 | 7 (24%) | 2 (8%) | 2·04 (0·77, 5·42); 0·15 | 3·63 (1·21, 10·90); 0·02 |
| Mild disability | | 8 (28%) | 4 (15%) | | |
| No cognitive disability | | 6 (21%) | 5 (19%) | | |
| ***Adjustments*** | | | | | |
| Adj. for centre | 27:24 | *69·33 (30·06)* | *53·68 (35·70)* | 13·76 (-4·45, 31·97); 0·14 | 22·0 (5·7, 38·3); 0·009 |
| Adj. for centre *(binary: Bristol vs. other)* | 27:24 | *69·33 (30·06)* | *53·68 (35·70)* | 14·54 (-3·78, 32·87); 0·12 | 23·19 (6·35, 40·04); 0·008 |
| Adj. for maternal education *(post-hoc)* | 27:23 | *69·33 (30·06)* | *55·90 (34·77)* | 11·50 (-6·86, 29·87); 0·21 | 20·08 (2·96, 37·21); 0·02 |
| Adj. for imbalance | 27:24 | *69·33 (30·06)* | *53·68 (35·70)* | 24·58 (6·69, 42·46); 0·008 | - |
| ***Multiple imputation[c]*** | | | | | |
| Assumption 1[d] | 36:33 | *65·24 (5·63)* | *50·81 (6·23)* | 14·43 (-2·10, 30·96); 0·09 | 21·17 (5·66, 36·68); 0·008 |
| Assumption 2[e] | 36:33 | *65·42 (5·45)* | *50·87 (6·31)* | 14·54 (-1·98, 31·07); 0·08 | 21·42 (6·21, 36·64); 0·007 |
| Assumption 3[f] | 36:33 | *62·95 (5·80)* | *49·41 (6·44)* | 13·55 (-3·84, 30·93); 0·12 | 20·53 (4·49, 36·56); 0·013 |
| Assumption 4[g] | 36:33 | *62·80 (5·91)* | *49·58 (6·47)* | 13·22 (-4·49, 30·93); 0·14 | 20·08 (3·79, 36·38); 0·017 |
| Assumption 5[h] | 34:32 | *66·85 (5·42)* | *53·70 (6·43)* | 13·14 (-3·67, 29·96); 0·12 | 20·47 (4·62, 36·31); 0·012 |
| ***Case scenarios (for those with unknown survival status)*** | | | | | |
| Best case scenario[i] | 31:26 | 65·04 (32·94) | 49·55 (37·22) | 15·49 (-3·13, 34·12); 0·101 | 20·67 (3·68, 37·65); 0·018 |
| Worst case scenario[j] | 31:26 | 60·38 (36·62) | 49·55 (37·22) | 10·83 (-8·83, 30·50); 0·274 | 15·28 (-3·72, 34·29); 0·113 |

[a]adjusted for sex, birthweight & grade of IVH
[b]giving children who have died post 2 years a cognitive quotient (CQ) score of 0
[c]standard errors replace standard deviations here
[d]imputing CQ for those who died post 2 years or were lost to follow up with no indicator for death
[e]imputing CQ for those who died post 2 years or were lost to follow up with an indicator for death
[f]imputing CQ for those who were lost to follow-up with no indicator for death and replace CQ with 0 for those who died post 2 years
[g]imputing CQ for those who were lost to follow-up with an indicator for death and replace CQ with 0 for those who died post 2 years
[h]imputing CQ for those who were lost to follow-up only with no indicator for death
[i]assuming the 2 children in the DRIFT group were all alive and without severe cognitive disability (with the median score for their group) at 10 years
[j]assuming the 2 children in the DRIFT group had died

**eTable 2. Subgroup analyses**

| | N (D:S) | Subgroup specific | | Interaction (95% C.I.); P value | Interaction[a] (95% C.I.); P value |
|---|---|---|---|---|---|
| | | DRIFT *Mean(SD)* | Standard *Mean(SD)* | | |
| ***Cognitive Quotient scores at school age (treatment-subgroup interaction)*** | | | | | |
| Gestation (<28 weeks) | 15:10 | 62·5 (28·4) | 42·9 (30·7) | 3·20 (-33·7, 40·1); 0·892 | -18·9 (-54·7, 17·0); 0·295 |
| Gestation (≥28 weeks) | 12:14 | 77·8 (31·1) | 61·4 (38·1) | | |
| Grade of IVH (Grade 3) | 14:13 | 75·6 (26·1) | 71·1 (36·3) | 25·0 (-9·2, 59·2); 0·149 | 15·7 (-19·8, 51·1); 0·379 |
| Grade of IVH (Grade 4) | 13:11 | 62·6 (33·6) | 33·1 (22·0) | | |
| Age[b] (<21 days) | 15:15 | 75·2 (30·1) | 60·7 (37·3) | -5·5 (-42·9, 32·0); 0·770 | -5·0 (-38·3, 28·2); 0·762 |
| Age[b] (≥21 days) | 12:9 | 62·0 (29·6) | 42·0 (31·3) | | |
| Unilateral dilatation[c] | 4:4 | 64·0 (23·7) | 35·4 (16·9) | 15·7 (-35·4, 66·8); 0·539 | 6·8 (-40·0, 53·6); 0·771 |
| Bilateral dilatation[c] | 23:20 | 70·3 (31·4) | 57·3 (37·6) | | |
| Sex: Male | 22:15 | 65·3 (31·6) | 47·6 (34·4) | -5·3 (-47·7, 37·2); 0·803 | -3·6 (-42·1, 34·8); 0·851 |
| Sex: Female | 5:9 | 86·9 (12·5) | 63·9 (37·6) | | |
| Pre enhanced vigilance[d] | 22:23 | 67·7 (33·1) | 51·5 (34·8) | -43·5 (-117·8, 30·9); 0·246 | -15·2 (-84·5, 54·0); 0·660 |
| Post enhanced vigilance[d] | 5:1 | 76·7 (6·4) | 104·0 (0) | | |
| Maternal educ. (Low)[e] | 10:11 | 64·2 (37·8) | 52·1 (31·8) | -0·9 (-39·0, 37·1); 0·961 | -15·8 (-50·8, 19·1); 0·366 |
| Maternal educ. (High)[e] | 17:12 | 72·4 (25·3) | 59·4 (38·4) | | |

[a]adjusted for sex, birthweight & grade of IVH,
[b]age at randomization,
[c]dilatation on ultrasound scan at randomization,
[d]in 2006 the trial was temporarily halted as there were concerns about secondary hemorrhages in the DRIFT group, after this time 7 more patients were recruited during an ‘enhanced vigilance’ period [5]
[e]Maternal education was collected at 10 years and therefore only classed as an indicator of education at baseline. This was classed as ‘low’ if the mother left school at 16 and ‘high’ if the mother carried on with further education post 16 and/or went to university.



Luyt K, *et al. Arch Dis Child Fetal Neonatal Ed* 2020; 105:466–473. doi: 10.1136/archdischild-2019-318231

**REFERENCES**

1. Whitelaw, A., et al., *Randomized Trial of Drainage, Irrigation and Fibrinolytic Therapy for Premature Infants with Posthemorrhagic Ventricular Dilatation: Developmental Outcome at 2 years.* Pediatrics, 2010. **125**(4): p. E852-E858.
2. Elliot, C. and P. Smith, *British Ability Scales 3rd edition*. 2012, London GL Assessment.
3. Bayley, N., *Bayley Scales of Infant & Toddler Development 3rd edition*. The Psychological Corporation: San Antonio, TX.
4. Biering, K., N.H. Hjollund, and M. Frydenberg, *Using multiple imputation to deal with missing data and attrition in longitudinal studies with repeated measures of patient-reported outcomes.* Clin Epidemiol, 2015. **7**: p. 91-106.
5. Whitelaw, A., et al., *Randomized clinical trial of prevention of hydrocephalus after intraventricular hemorrhage in preterm infants: Brain-washing versus tapping fluid.* Pediatrics, 2007. **119**(5): p. E1071-E1078.
